# Afterhyperpolarization (AHP) regulates the frequency and timing of action potentials in the mitral cells of the olfactory bulb: role of olfactory experience

**DOI:** 10.14814/phy2.12344

**Published:** 2015-05-27

**Authors:** Maël Duménieu, Nicolas Fourcaud-Trocmé, Samuel Garcia, Nicola Kuczewski

**Affiliations:** Centre de Recherche en Neurosciences de Lyon, INSERM U1028/CNRS UMR5292, Université Lyon1Lyon, France

**Keywords:** Firing, intrinsic plasticity, recurrent synaptic transmission

## Abstract

Afterhyperpolarization (AHP) is a principal feedback mechanism in the control of the frequency and patterning of neuronal firing. In principal projection neurons of the olfactory bulb, the mitral cells (MCs), the AHP is produced by three separate components: classical potassium-mediated hyperpolarization, and the excitatory and inhibitory components, which are generated by the recurrent dendrodendritic synaptic transmission. Precise spike timing is involved in olfactory coding and learning, as well as in the appearance of population oscillatory activity. However, the contribution of the AHP and its components to these processes remains unknown. In this study, we demonstrate that the AHP is developed with the MC firing frequency and is dominated by the potassium component. We also show that recurrent synaptic transmission significantly modifies MC AHP and that the strength of the hyperpolarization produced by the AHP in the few milliseconds preceding the action potential (AP) emission determines MC firing frequency and AP timing. Moreover, we show that the AHP area is larger in younger animals, possibly owing to increased Ca^2+^ influx during MC firing. Finally, we show that olfactory experience selectively reduces the early component of the MC AHP (under 25 msec), thus producing a modification of the AP timing limited to the higher firing frequency. On the basis of these results, we propose that the AHP, and its susceptibility to be selectively modulated by the recurrent synaptic transmission and olfactory experience, participate in odor coding and learning by modifying the frequency and pattern of MC firing.

## Introduction

Feedback mechanisms are an important component in the control of neuronal firing. Feedback mechanisms are directly elicited by the action potentials (APs) generated by a neuron, exerting a rapid and dynamic control on consequent AP emissions and affecting both the frequency and the patterning of neuronal firing. One of the most common neuronal feedback processes is the afterhyperpolarization (AHP) that follows an AP emission. This repolarization of the membrane potential, classically generated by the opening of calcium- and voltage-dependent potassium channels, reduces the duration of the AP and defines a time-window refractory to the emission of a new AP (Sah and Faber, 2002; Faber and Sah, 2007) participating, in this way, in shaping AP patterns. The importance of the AHP in neuronal processing is supported by the fact that its dysregulation is involved in brain pathologies such as epilepsy (Behr et al. 2000) and cognitive impairment (Moyer et al. 1992).

Mitral cells (MCs) are the principal neurons of the olfactory bulb (OB), which transmit odor information to higher-order cortices, thus ultimately reflecting the odor processing in the OB via their firing (Bathellier et al. 2010). Accumulating evidences suggest that the coding of olfactory information relies on precise temporal pattern of MCs firing (Gschwend et al. 2012; Uchida et al. 2014) whereas spike correlations within specific MC subpopulations allow the transfer of olfactory information from the OB to higher-order cortices (Laurent 2002). Interestingly, it has been shown that olfactory learning leads to fine regulation of MC spike synchronicity (Doucette et al. 2011). Precise control of the temporal patterning of MCs APs appears therefore to be required for olfactory coding and learning in the OB. However, the cellular mechanisms regulating the timing of APs have not been fully elucidate and the AHP could be one of those. In MCs, spiking triggers the classical potassium AHP (Maher and Westbrook 2005), as well as a double-component synaptic feedback mechanism sequence as follows: first, AP propagation in the dendrites elicit glutamate release which produces a feedback autoexcitation of MCs known as recurrent dendrodendritic excitation (DDE) (Aroniadou-Anderjaska et al. 1999; Friedman and Strowbridge 2000; Salin et al. 2001); second, dendritic glutamate release can activate the dendritic spines of local interneurons, producing a GABA-mediated feedback inhibition of MC, termed recurrent dendrodendritic inhibition (DDI) (Isaacson and Strowbridge 1998; Schoppa et al. 1998). Ultimately, the MC AHP potentially results from three feed-back components: potassic, glutamatergic, and GABAergic. While a large number of studies have elucidated the cellular mechanisms involved in the recurrent synaptic transmission, less attention has been paid to the potassic AHP of MCs. Moreover, results on the cellular mechanisms involved in the recurrent synaptic transmission have been drawn from the isolation of each component. A study considering the relative contribution of the three feedback processes to the AHP is necessary to better understand their relative impact upon MC firing.

In the present report, we investigate the impact of the AHP on MC action potential timing and the contribution of animal age, olfactory experience and of the different AHP components on this process.

## Materials and Methods

Animal handling was conducted in accordance with the European Community Council Directive 86/609/EEC, with the approval of the ethics committee CEEA-55 of the “Université Claude Bernard-Lyon1”. Experiments were performed on P13-P16 or P36-P40 male Long Evans rats (Janvier, Le Genest-Saint-Isle, France). The animals were maintained on a normal light cycle with water and food ad libitum.

### Slice preparation

Animals were anaesthetized with an intraperitoneal injection of ketamine (50 mg/mL) and decapitated. The head was quickly immersed in ice-cold (2–4°C) carbogenized artificial cerebrospinal fluid (cACSF; composition: 125 mmol/L NaCl, 4 mmol/L KCl, 25 mmol/L NaHCO_3_, 0.5 mmol/L CaCl_2_, 1.25 mmol/L NaH_2_PO_4_, 7 mmol/L MgCl_2_, and 5.5 mmol/L glucose; pH = 7.4) oxygenated with 95% O_2_, 5% CO_2_. The osmolarity was adjusted to 320 mOsm with sucrose. OBs were removed and cut in horizontal slices (400 *μ*m thick) using a Leica VT1000s vibratome (Leica Biosystems, Nanterre, France). Slices were incubated in Gibb's chamber at 30 ± 1°C using ACSF similar to the cACSF, except that CaCl_2_ and MgCl_2_ concentrations were 2 and 1 mmol/L, respectively.

### Odor exposure

From P29 to P35, the rats were exposed to isoamyl acetate 20 min/day for 7 consecutive days. The odor was presented in a tea ball containing an odorized pellet. We empirically set the odor concentration by adjusting the quantity of the odorized pellet until the odor could be detected from a distance of approximately 20 cm from the investigator's nose. Exposed animals were used for electrophysiological experiments between 1 to 3 days after the last odor exposure.

### Electrophysiological recordings

Slices were transferred into a recording chamber mounted on an upright microscope (Axioskop FS, Zeiss, Marly le Roi, France) and perfused with oxygenated ACSF (4 mL/min) at 30 ± 1°C. Neurons were visualized using a 40× objective and a Hamamatsu “Orca Flash 4.0” camera. Measurements were performed with an RK 400 amplifier (BioLogic, Claix, France). The data were acquired with a sampling frequency of 25 kHz on a PC-Pentium D computer using a 12-bit A/D-D/A converter (Digidata 1440A, Axon Instruments, Union City, GA) and PClamp10 software (Axon Instruments). The junction potential was corrected offline. Patch-clamp configurations were achieved with borosilicate pipettes (o.d.: 1.5 mm; i.d.: 1.17 mm; Clark Electromedical Instruments, Edenbridge, UK). The recording pipette was filled with the following intracellular solution (131 mmol/L K-gluconate, 10 mmol/L HEPES, 1 mmol/L EGTA, 1 mmol/L MgCl_2_, 2 mmol/L ATP-Na_2_, 0.3 mmol/L GTP-Na_3_, and 10 mmol/L phosphocreatine; pH = 7.3, 290 mOsm). In our experimental conditions, the equilibrium potential of chloride ions (E_Cl_) was −110 mV, and that of potassium ions (E_k_) was −92 mV. Mitral cells were recorded in the current-clamp configuration and kept at a membrane potential of −63 mV by steady current injection. To isolate the different components of the AHP, ionotropic receptor antagonists were diluted in ACSF. GABA_A_ receptors were blocked with gabazine (GBZ) (10 *μ*mol/L, Tocris Bioscience, Lille, France). NMDA (N-methyl-d-aspartate)-type ionotropic glutamate receptors were blocked with 50 *μ*mol/L D-APV (d-(−)-2-amino-5-phosphonopentanoic acid, Tocris Bioscience), and AMPA (alpha-amino-3-hydroxy-5-methyl-ioxazole-4-propionic acid)-type receptors were blocked with 10 *μ*mol/L NBQX (2,3-dioxo-6-nitro-1,2,3,4-tetrahydrobenzo[f]quinoxaline-7-sulfonamide, Tocris Bioscience).

### Ca^2+^ imaging

Real-time Ca^2+^ imaging was performed on MC soma using an epifluorescence microscope (Zeiss axioscope) with a 40× objective (Zeiss Plan-APOCHROMAT). Ca^2+^-induced fluorescence was detected with excitation (470/40 nm) and emission (dichroic mirror: 495; 525/50 nm) band pass filters (Zeiss filter set 38 HE). The illumination was produced by a white LED (Dual Port OptoLED, CAIRN, Faversham, UK) using the same illumination power for the different samples. Pictures were captured with a digital CCD camera (ORCA Flash 4.0, Hamamatsu Photonics, Maccy, France). Frames for time-lapse imaging were acquired (every 20 msec) using HCimage software (Hamamatsu Photonics, Massy, France) and analyzed using ImageJ software (NIH, Bethesda, MD). The Ca^2+^ probes Fura-2 (200 *μ*mol/L) or Fura2-FF (400 *μ*mol/L) were dissolved in the intracellular medium. After passing in whole-cell configuration, 10 (Fura2) or 20 min (Fura2-FF) was allowed to pass before image acquisition. The ΔF/F was calculated as follows: The basal fluorescence (F_bas_) was obtained by averaging the neuronal fluorescence intensity of the 500 msec preceding MC intracellular stimulation. The frame fluorescence (F_fr_) was the neuronal fluorescence intensity of the frame. Therefore, ΔF/F = (F_fr_ − F_bas_)/F_fr_. The data are expressed as the mean ± SEM.

### Data analysis

Data analysis was performed using OpenElectrophy (Garcia and Fourcaud-Trocmé 2009), SciPy, and MySql database software (open source licenses). All single-traces were visually inspected to eliminate abrupt Vm modifications contaminating the signal. To reduce the effect produced by the spontaneous modification of the resting Vm on the AHP (i.e., due to the modification of the driving forces), all the traces for which the Vrest was not in the range from −64 to −62 mV were excluded from the analysis. For the AHP measurements, the baseline, which was calculated as the median of the 5 msec preceding the first step, was subtracted from each trace. Then, the AHPs were aligned on the crossing zero of the AP repolarizing phase and averaged (8–15 traces). The AHP area was calculated as the area between the baseline and the time to which the AHP returned to 10% of the AHP peak. The different components of the AHP were calculated by offline subtraction of the average signals obtained in the different conditions (control, GBZ, and GBZ+NBQX+APV). The Spike timing analysis was performed by calculating the latency of the action potential peak from the beginning of the current step injected to generate the spike. An average of 8–15 measurements was taken as representative of the latency for each condition. Unless otherwise specified, data are expressed as the average ± SEM. Statistical comparisons were performed using various parametric and nonparametric tests, depending on the normality of value distributions as follows: the Mann–Whitney test for independent samples, the Wilcoxon signed-rank test for paired samples, paired or unpaired Student's *t*-test and the Friedman test for multiple comparisons. Moreover, correlation was assessed by the Pearson or Spearman test. For all the tests, a *P* < 0.05 was indicated by *, *P* < 0.01 by **, and *P* < 0.001 by ***.

## Results

### The MC AHP increases with the neuronal firing and decrease with the animal age

MCs were recorded in current-clamp configuration from OB slices of P36-P40 rats (P38 group) and P13-P16 rats (P15 group). The average resting membrane potential (V_rest_) was similar for the two groups (−62 ± 4 mV for P38, *n* = 32; −63 ± 4 mV for P13, *n* = 13, *P* = 0.53, unpaired *t*-test; average ± SD). However, the membrane resistance was lower in the P15 group (116 ± 33 MΩ at P15 and 146 ± 57 MΩ at P38; *P* = 0.02, unpaired *t*-test) whereas the membrane capacitance was higher (Cm: 134 ± 11 pF at P15 and 87 ± 6 pF at P38; *P* = 0.001, unpaired t-test), suggesting a reduction in the MC surface with increasing animal age. In order to have similar experimental conditions for all recorded neurons the V_rest_ was maintained at −63 ± 1 mV by steady current injection (V_hold_).

**Figure 1:**
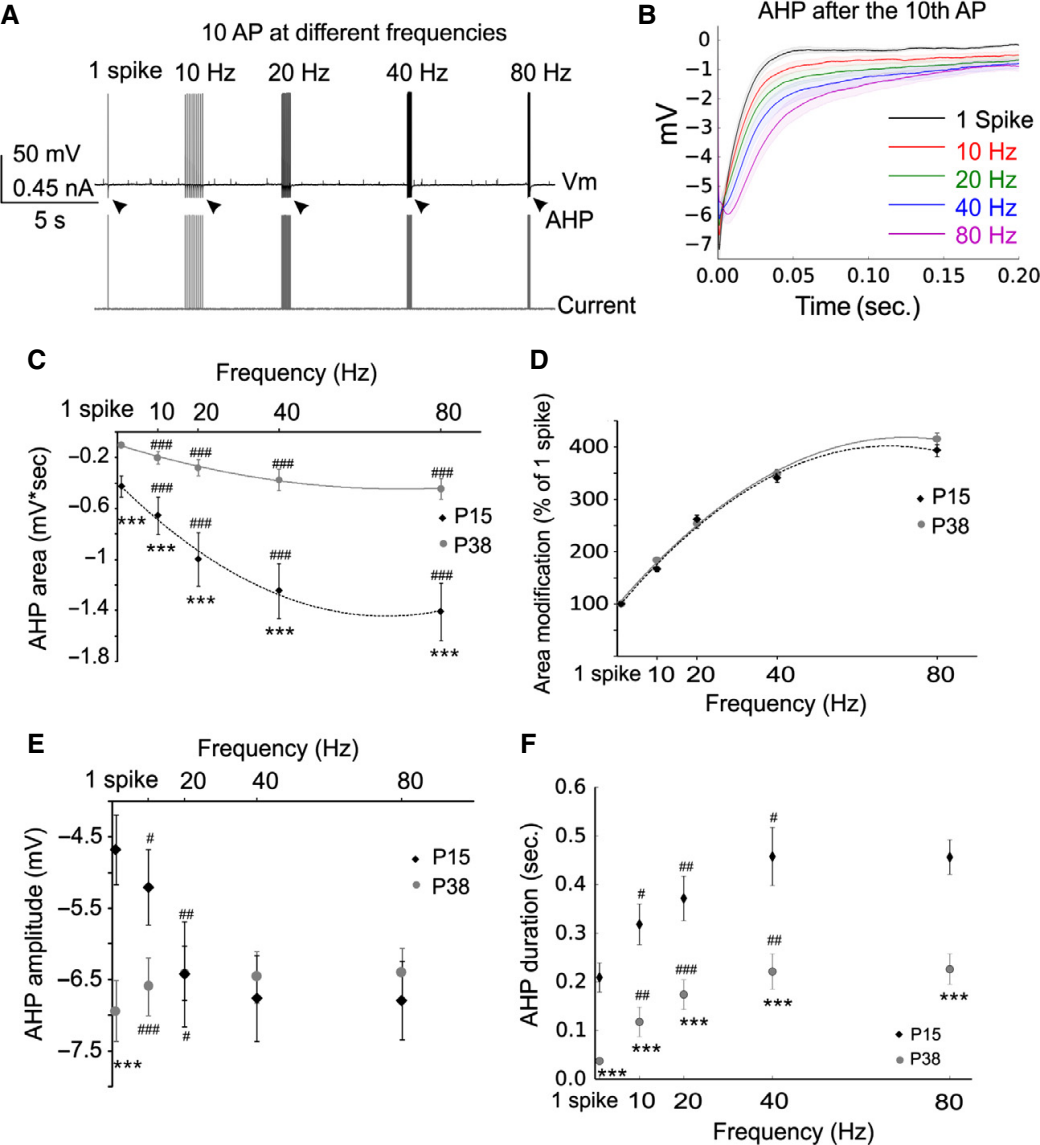
The global MC AHP increases with the firing frequency. (A) Schematic representation of the stimulating protocol used to elicit MC firing at different frequencies. Black arrows indicate the AHPs following the last spike of each train that were used for analysis. (B) Average traces of the AHP following the last spike of the train at different frequencies (from the P38 group). Shadow areas represent the SEM. (C) The AHP area is significantly larger in MC from the P15 group and increases with the MC firing frequency for all the frequencies tested at both ages. (D) Normalization of the AHP area to that produced by 1 spike show a similar frequency-dependent increase in the AHP between the two age groups. The AHP amplitude decreases with MC firing frequency, in the range from 1 spike – 20 Hz, in MC from older rats but increase in the same frequency range in MC from the P15 group. (F) AHP duration is also larger in the P15 group and increases with the firing frequency in the range 1-spike 40 Hz. ^#^Significantly differs from the previous frequency. *Significantly difference between the groups.*P < 0.05 **P < 0.01; ***P < 0.001.

Firing of MCs generated by long current step injection is highly heterogeneous between MCs population with some neurons presenting clustered bursting activity (Balu et al. 2004; Padmanabhan and Urban 2010; Fadool et al. 2011). This characteristic makes difficult to finely characterize the MCs AHP based on the hyperpolarization produced at the end of a long depolarizing current step. To overcome this limitation, MC firing was produced by short depolarizing current injections (2 msec, 30–80 nA). Four bursts of ten APs at different frequencies (10, 20, 40, and 80 Hz) were induced following the generation of a single AP (Fig.1A) and the AHP measured after the last APs of each burst. Figure1B shows the average traces of the AHP produced by the different firing frequencies in the P38 group. Under these conditions, a significant frequency-dependent increase in the AHP area was observed for the two groups (Wilcoxon signed-rank test); moreover, the AHP area was significantly bigger in younger rats (Fig.1C, Table1, Mann–Whitney). To determine whether the frequency-dependent increase in the AHP area has a similar evolution in the two groups, we normalized, for each cell, the AHP areas obtained from the different firing frequencies to that of 1 spike. As shown in Figure1D, no differences were observed between the two ages.

**Table 1:** AHP areas compared by MC firing frequency and animal age.

Frequency	1 spike	10 Hz	20 Hz	40 Hz	80 Hz	Friedman between frequencies
P15 area (mV^*^sec)	0.4 ± 0.08	0.65 ± 0.14	0.99 ± 0.21	1.24 ± 0.22	1.40 ± 0.22	2^*^10^−16^
P38 area (mV^*^sec)	0.1 ± 0.01	0.2 ± 0.05	0.28 ± 0.06	0.37 ± 0.08	0.44 ± 0.08	2^*^10^−16^
P15 *P*-value to previous frequency		**<0.001**	**<0.001**	**<0.001**	**<0.001**	
P38 *P*-value to previous frequency		**<0.001**	**<0.001**	**<0.001**	**<0.001**	
*P*-value (P15 vs. P38)	**<0.001**	**<0.001**	**<0.001**	**<0.001**	**<0.001**	

Bold indicates statistically significant difference.

A difference in the AHP amplitude between P15 and P38 group was observed only for one spike. Interestingly, while the AHP amplitude significantly increases with the MCs firing frequency in the P15 group, it decreases in the P38 animals. For this reason, the AHP amplitude was similar between the two groups for frequencies higher than 1 spike (Fig.1E, Table2, Mann–Whitney). An age-dependent increase in AHP duration and frequency was observed (Fig.1F, Wilcoxon signed-rank test for frequency and Mann–Whitney test for age comparisons). These results suggest that the increase in the AHP area that is produced by an increase in MC firing frequencies is mainly due to the increase in the AHP duration, and that the greater AHP area observed in younger animals is also due to greater AHP duration in those animals.

**Table 2:** AHP amplitudes compared by MC firing frequency and animal age.

Frequency	1 spike	10 Hz	20 Hz	40 Hz	80 Hz	Friedman between frequencies
P15 amplitude (mV)	−4.7 ± 0.5	−5.2 ± 0.5	−6.4 ± 0.7	−6.8 ± 0.6	−6.8 ± 0.5	**0.04**
P38 amplitude (mV)	−7.1 ± 0.4	−6.6 ± 0.4	−6.4 ± 0.4	−6.5 ± 0.4	−6.4 ± 0.3	0.6
P15 *P*-value to previous frequency		**0.03**	**0.005**	0.29	0.89	
P38 *P*-value to previous frequency		**<0.001**	**0.015**	0.61	0.53	
*P*-value (P15 vs. P38)	**<0.001**	0.05	0.98	0.65	0.54	

Bold indicates statistically significant difference.

### Impact of potassic, GABAergic, and glutamatergic components on MC AHP

To determine the relative contribution of the recurrent DDI, DDE, and potassium components on the AHP and to determine whether such contribution depends on the MC firing frequency and animal age, we isolated these components from the AHP using a pharmacological approach.

**Figure 2:**
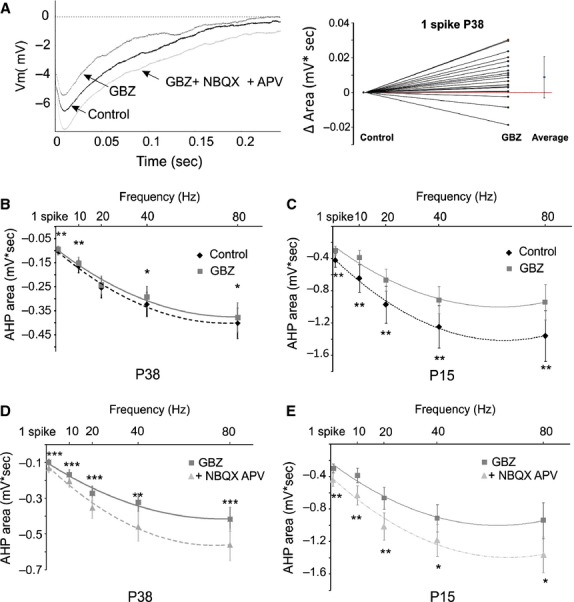
The recurrent dendrodendritic synaptic transmission participates in the AHP. (A) Left, representative traces showing the effect of antagonists of ionotropic synaptic transmission (GBZ and NBQX/APV) on the MC AHP. Right, modification of the AHP area produced by GBZ application (average ± SD). (B) The GABA_A_ antagonist GBZ reduced the AHP area for all the tested frequencies, except for 20 Hz, demonstrating the presence of a recurrent DDI in MC of the P38 group (*n* = 23). (C) GBZ significantly reduces the AHP area in the P15 group (*n* = 12). (D and E) NBQX and APV application in the presence of GBZ increased the residual AHP in both P15 (*n* = 10) and P38 (*n* = 18) groups, demonstrating the presence of a recurrent DDE. *Significant difference between the two conditions for each frequency. *P < 0.05, **P < 0.01, ***P < 0.001

**Figure 3:**
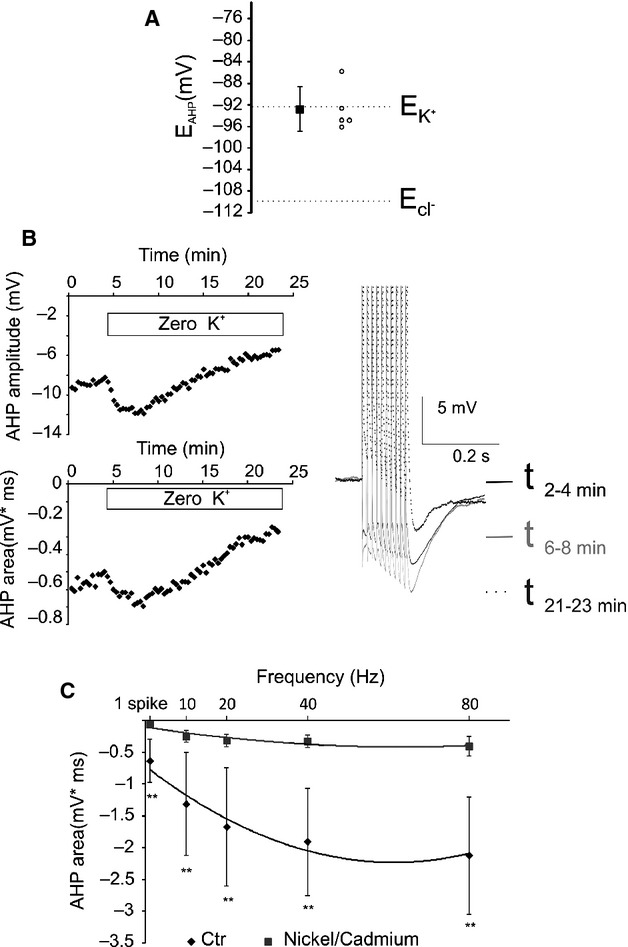
The residual component of the AHP is mediated by K^+^ currents. (A) The reverse potential of the residual AHP is close to the calculated Ek. Empty circles represent the E_AHP_ of single neurons, whereas the black squares represent the average value of E_AHP_ ± SEM. (B) Removal of the extracellular K^+^ produced a transient increase, followed by a reduction, in the residual AHP. This specific pattern is possibly caused by the consecutive transient increase and sustained decrease in the potassium driving force due to the processes of extracellular and then intracellular potassium depletion. (C) The residual component of the AHP is reduced by the Ca^2+^ channel antagonists Ni^2+^/Cd^2+^. *Significant difference between the two conditions for each frequency. **P < 0.01.

First, we applied the GABA_A_ receptor antagonist gabazine (GBZ) (10 *μ*mol/L) to block and determine the recurrent DDI. Subsequently, glutamate antagonists – NBQX (10 *μ*mol/L) and D-APV (40 *μ*mol/L) – were applied in the presence of GBZ to determine the recurrent DDE (Fig.2A left). Bath application of GBZ produced a small but significant decrease in the AHP for all tested frequencies in the P38 group except for 20 Hz (Fig.2A right, Fig.2B; Table3, Wilcoxon signed-rank test), and a more consistent reduction in the AHP area in the P15 group (Fig.2C; Table3, Wilcoxon signed-rank test) indicating the participation of the recurrent DDI to the MC AHP. Therefore, even if the recurrent DDI participates in the AHP, a relevant portion of the postspike inhibition does not rely on GABA_A_ transmission. Bath application of NBQX (10 *μ*mol/L) and D-APV (40 *μ*mol/L) in the presence of GBZ produced a significant increase in the residual AHP area for all tested frequencies and for the AHP produced by a single AP in both groups of age (Fig.2D and E; Table3, Wilcoxon signed-rank test). This suggests the recurrent DDE modulates the AHP of the MCs by reducing the postspike hyperpolarization. In the presence of GABA_A_ and glutamate ionotropic receptors antagonists, the residual AHP component reversed at the membrane potential close to E_K_ (E_AHP_ = −92.7 ± 4 mV SD, *n* = 5; calculated E_K_ = −92.3 mV, Fig.3A). Moreover, following the removal of the extracellular potassium, the residual component transiently increased for a few minutes and decreased thereafter (Fig.3B, *n* = 3), as expected for a transient increase in the K^+^ driving force followed by intracellular K^+^ depletion. These results suggest that the residual AHP is mediated by K^+^ channels. Finally, the potassium component was significantly reduced by applying the SK blocker apamin (10–100 nmol/L) (AHP area = 73 + 7% of CTR at 40 Hz, *P* < 0.05, Wilcoxon signed-rank test, *n* = 5, data not shown) or by the Ca^2+^ channel blocker Ni (5 mmol/L, *n* = 4) or Cd (250 *μ*mol/L, *n* = 4) (AHP area 35 ± 9% of CTR at 40 Hz, *P* < 0.01, Wilcoxon signed-rank test *n* = 8; Fig.3C), demonstrating that the global MC AHP is primarily due to the activation of Ca^2+^-dependent potassium channels (Sah and Faber 2002).

**Table 3:** Effects of GABA_A_ and ionotropic glutamatergic receptors antagonists on MC AHP area. Comparisons at different MC firing frequencies and animals age.

Frequency	1 spike	10 Hz	20 Hz	40 Hz	80 Hz
P38 area (mV^*^sec) in GBZ (*n* = 21)	0.09 ± 0.01	0.15 ± 0.02	0.24 ± 0.04	0.29 ± 0.04	0.37 ± 0.06
P38 *P*-value compared to CTR	**0.002**	**0.007**	0.12	**0.01**	**0.04**
P38 area (mV^*^sec) in NBQX APV (*n* = 17)	0.13 ± 0.02	0.2 ± 0.03	0.35 ± 0.06	0.45 ± 0.08	0.56 ± 0.09
P38 *P*-value compared to GBZ	**0.004**	**<0.001**	**0.002**	**<0.001**	**<0.001**
P15 area (mV^*^sec) in GBZ (*n* = 12)	0.32 ± 0.05	0.43 ± 0.09	0.71 ± 0.12	0.95 ± 0.14	1.03 ± 0.16
P15 *P*-value compared to CTR	**0.002**	**0.009**	**0.002**	**0.002**	**0.004**
P15 area (mV^*^sec) in NBQX APV (*n* = 11)	0.43 ± 0.08	0.63 ± 0.11	1.01 ± 0.17	1.18 ± 0.20	1.36 ± 0.22
P15 *P*-value compared to GBZ	**0.003**	**0.003**	**0.001**	**0.02**	**0.01**

Bold indicates statistically significant difference.

**Figure 4:**
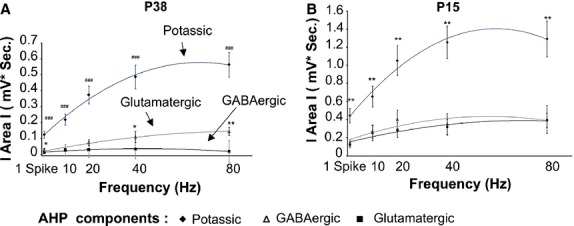
The area of the potassium component of the AHP predominates over the areas of the synaptic components. The absolute value of the areas of the different AHP components is depicted as a function of the MC firing rate. (A) P38 group; note that the potassium and the glutamatergic components but not the GABAergic component increase with the firing frequency (*P* < 0.001, Friedman). (B) P15 group, all component increases with the MC firing rate (*P* < 0.001, Friedman). *Significant difference between the glutamatergic and GABAergic components. ^#^Significant difference between the potassium and the synaptic components. *P < 0.05; **P < 0.01; ^###^*P* < 0.001.

To determine the relative contribution of the three feedback components to the AHP, we first determined the area of the different components by a subtraction approach (see methods), and plotted absolute values to facilitate their visual comparison (Fig.4). This approach indicated that, in both P38 and P15 groups, the potassium component predominates over the synaptic ones for all tested frequencies and for the AHP produced by a single AP (Fig.4, Tables4, 5, Wilcoxon signed-rank test). When considering the synaptic components of the AHP we found that, in the P38 group, the area of the DDE overcomes the recurrent DDI for the frequencies higher than 20 Hz (Fig.4A, Table1, Wilcoxon signed-rank test). Such an effect is produced because of the frequency-dependent increases in the glutamatergic (*P* = 7*10^−12^, Friedman), but not the GABAergic, component (*P* = 0.1, Friedman). A frequency-dependent increase in the potassium component is also present (p < 0.001, Friedman). On the other hand, in younger animals (P15) the synaptic components of the AHP are similar for the entire MC firing frequency tested (Fig.4B, Table5, Wilcoxon signed-rank test). Finally, the area of all the three AHP components were significantly higher in P15 compared to P38 animals (*P* < 0.05 for the whole frequency range, Mann–Whitney).

**Table 4:** AHP components in the P38 group. *P* values depict the statistical comparison between the different components: pot = potassium, glut = glutamatergic, gaba = GABAergic.

Frequency	Area (mV^*^sec)			*P* value		
	Potassium	GABAergic	Glutamatergic	p pot-glut	p pot-gaba	p glut-gaba
1 spike	0.12 ± 0.02	0.011 ± 0.003	0.029 ± 0.02	5^*^10^−**5**^	5^*^10^−**5**^	**0.04**
10 Hz	0.21 ± 0.03	0.028 ± 0.03	0.030 ± 0.03	5^*^10^−**5**^	2^*^10^−**4**^	0.21
20 Hz	0.37 ± 0.06	0.031 ± 0.05	0.074 ± 0.06	5^*^10^−**5**^	1^*^10^−**4**^	0.08
40 Hz	0.48 ± 0.07	0.035 ± 0.06	0.11 ± 0.07	5^*^10^−**5**^	6^*^10^−**5**^	**0.01**
80 Hz	0.56 ± 0.08	0.020 ± 0.07	0.14 ± 0.08	5^*^10^−**5**^	6^*^10^−**5**^	**0.006**

Bold indicates statistically significant difference.

**Table 5:** AHP components in the P15 group. *P* values depict the statistical comparison between the different components: pot = potassium, glut = glutamatergic, gaba = GABAergic.

Frequency	Area (mV^*^sec)			*P* value		
	Potassium	GABAergic	Glutamatergic	p pot-glut	p pot-gaba	p glut-gaba
1 spike	0.44 ± 0.08	0.12 ± 0.04	0.14 ± 0.04	**0.005**	**0.005**	0.95
10 Hz	0.65 ± 0.11	0.25 ± 0.08	0.27 ± 0.07	**0.005**	**0.005**	0.72
20 Hz	1.05 ± 0.16	0.28 ± 0.08	0.40 ± 0.10	**0.005**	**0.005**	0.20
40 Hz	1.25 ± 0.18	0.31 ± 0.09	0.35 ± 0.11	**0.005**	**0.005**	0.57
80 Hz	1.29 ± 0.20	0.39 ± 0.08	0.40 ± 0.15	**0.005**	**0.005**	0.95

Bold indicates statistically significant difference.

**Table 6:** Modifications of spike latency between the first and the last AP of each train at the different frequencies and animal age. The values are expressed as % of latency modification compared to the first AP.

Frequency	10 Hz	20 Hz	40 Hz	80 Hz	Friedman between frequencies (*P*)
P15	8 ± 2%	15 ± 3%	22 ± 3%	28 ± 3%	**<0.001**
P38	1 ± 0.4%	2 ± 0.8%	8 ± 2%	33 ± 3%	**<0.001**
*P*-value P15 vs. P38	**<0.001**	**<0.001**	**<0.001**	0.33	**###**

Bold indicates statistically significant difference.

**Figure 5:**
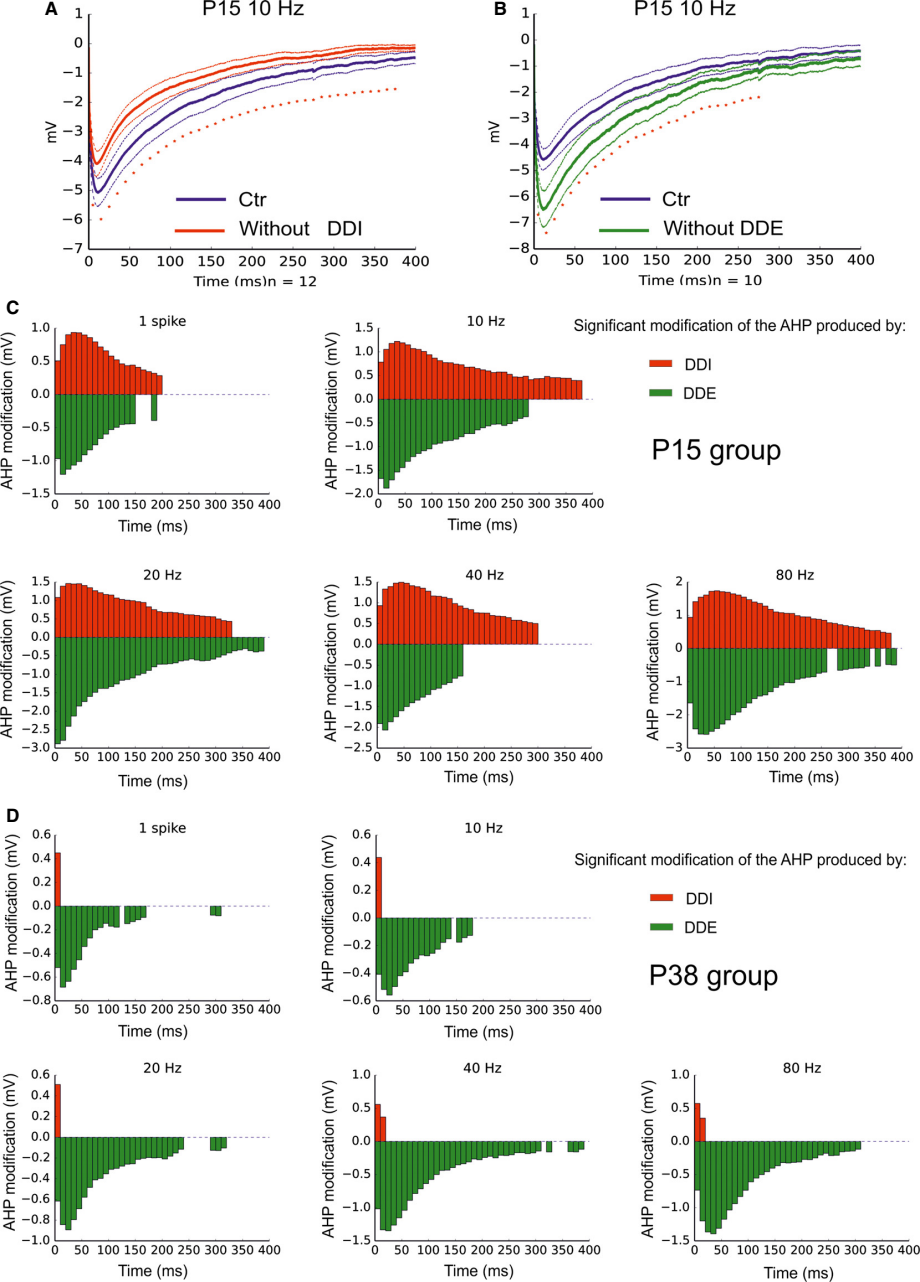
The impact of the recurrent DDE and DDI change with the animal age. (A) Average trace of the AHP in control condition (blue) and in the absence of recurrent DDI (red), that is, in the presence of GBZ. (B) Average trace of the AHP in control condition (blue) and in the absence of recurrent DDE (green), sees methods. Tin lines represent the SEM. Asterisks represent significant difference between the two curves (calculated on the average values of 10 msec bins). (C and D) Time course of AHP modification by the recurrent DDI (red) and DDE (green) in MC from P15 and P38 groups, respectively. Bars represent the difference between control and red curve (red) or control and blue curve (blue) for times bins that reach significance.

We next determined the impact of the synaptic components on the kinetic of the MC AHP. To determine the action of recurrent DDI on the AHP kinetic, we compared the AHP in control condition to the AHP observed in the presence of GBZ (Fig.5A) and calculated the differences between the two curves at different time bins (Fig.5C and D). The only time bins for which GBZ produce a significant difference are represented (red bars). The major difficulty to experimentally isolate the effect of recurrent DDE on MC AHP is the fact that the antagonists of glutamatergic transmission also block the recurrent DDI. To overcome this limitation, we reconstructed the AHP deprived of the recurrent DDE by subtracting the calculated glutamatergic component (see methods) from the traces of the control AHP. We then compared the control and DDE-deprived traces (Fig.5B–D). This analysis shows that in MC from P15 groups the recurrent DDI and DDE have a similar effect on the kinetics of the AHP. However, in older animals the action of the recurrent DDI was limited to the first 10–20 msec of the AHP while the recurrent DDE affect the AHP for several hundred milliseconds. Together, these results demonstrate that the potassium component is predominant in the MC AHP and that the impact of the recurrent synaptic transmission on MC AHP changes with the age of animal, with recurrent DDI capable of selectively enhancing the early, but not late, AHP in older animals.

### Role of Ca^2+^ influx in the frequency- and age-dependent modification of MC AHP

**Figure 6:**
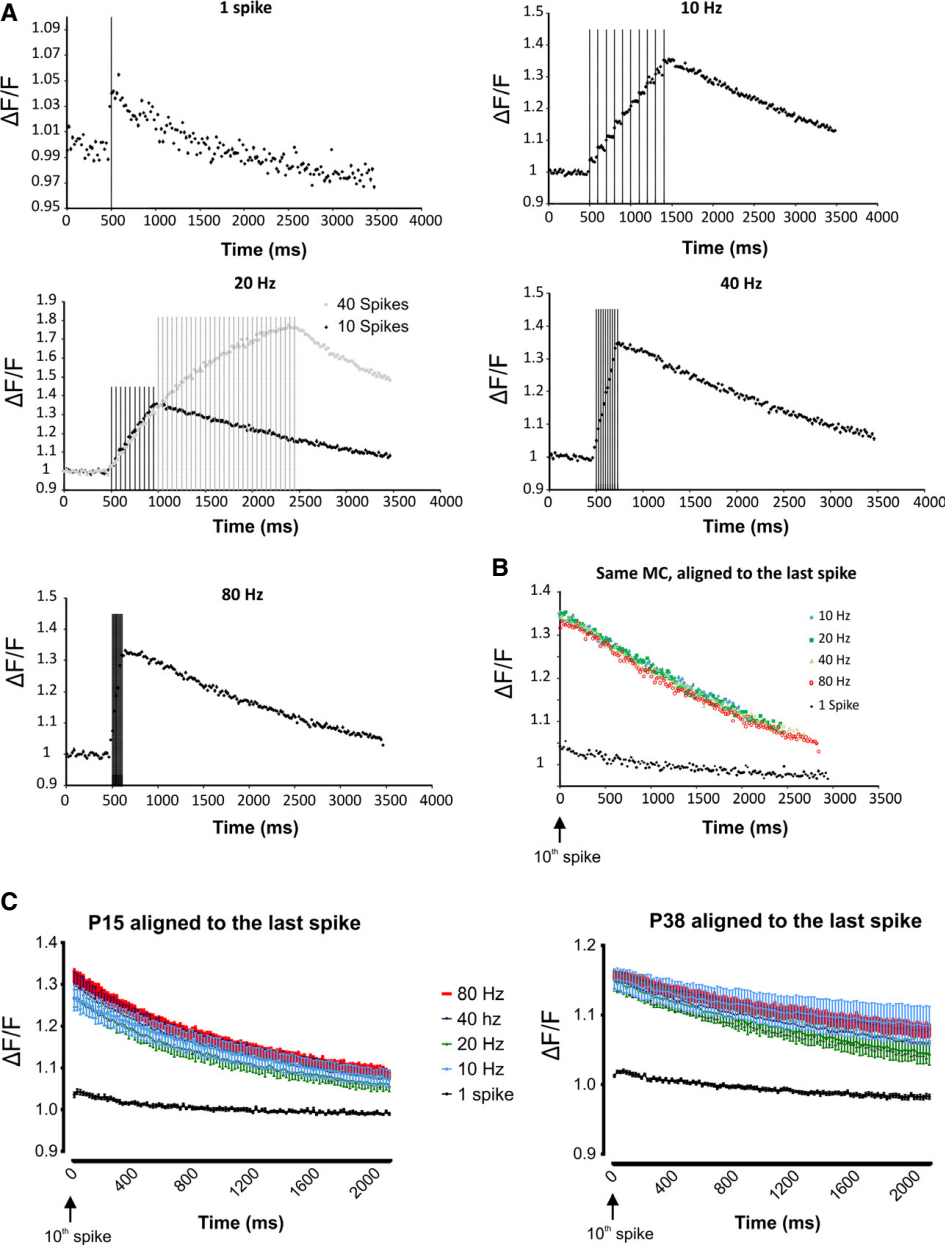
The intracellular somatic Ca^2+^ increase in the MC depends on the number of spikes but not on their firing frequency. (A) Representative example of the Ca^2+^ signal produced in a MC by the different firing frequencies. At 20 Hz, note that the Ca ^2+^ signal increases almost linearly when 40 spikes were generated, demonstrating that the Ca^2+^ influx produced by 10 spikes is below the saturation level of the Ca^2+^ probe used. The vertical lines represent the times of spike occurrence. (B) Same neuron, superposition of the ΔF/F at the end of the 10 spike train for the different firing frequencies; no obvious differences are observed. (C) Average of the ΔF/F at the end of the 10 spike train for the different firing frequencies for the P13 group (*n* = 13 MC) and for the P38 group (*n* = 14 MC). Within each group, no significant differences were observed between ΔF/F produced by the different firing frequencies. Error bars represent the SEM.

**Figure 7:**
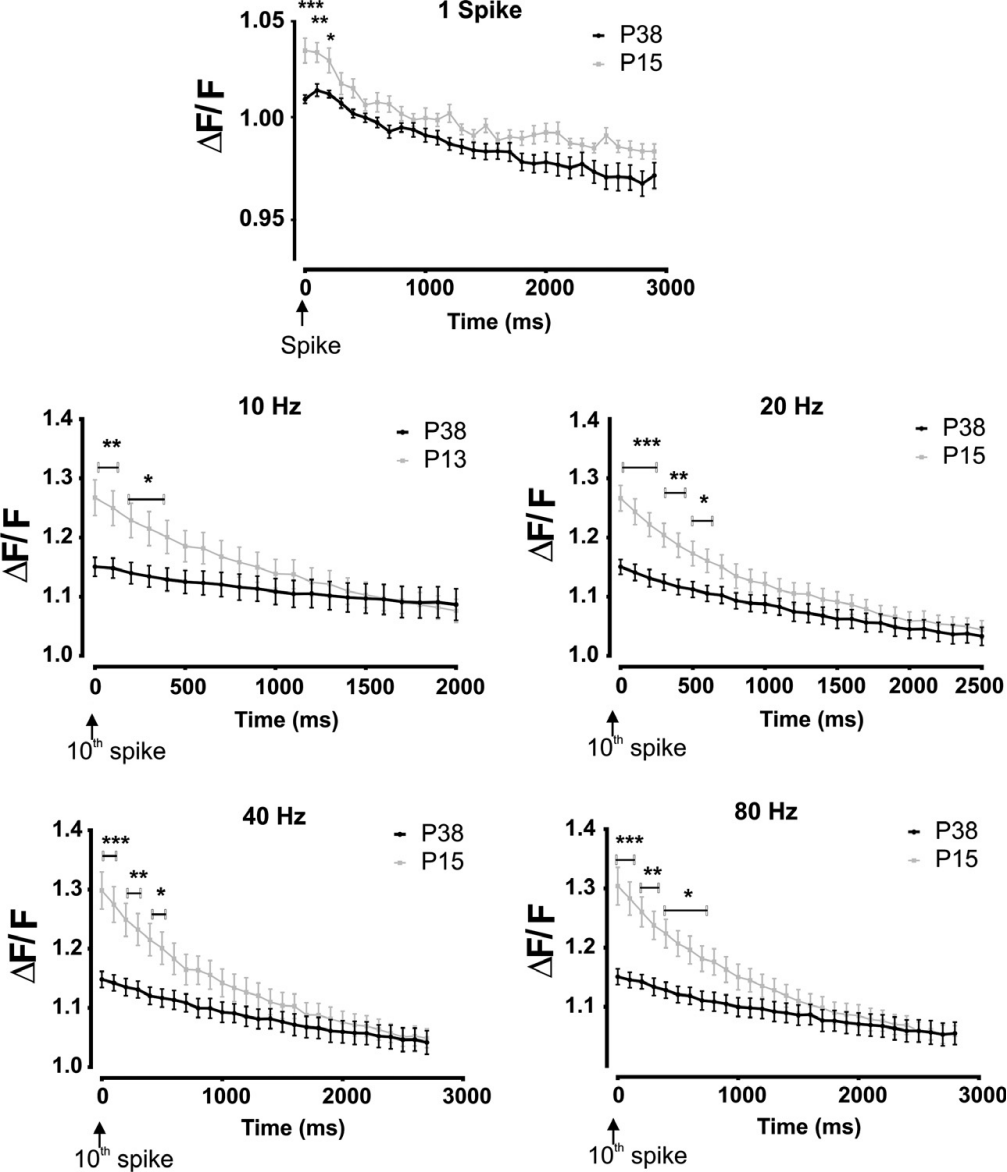
The spike-induced intracellular Ca^2+^ increase is larger in MCs from P13 than in MCs from P38 rats for the entire firing frequency range tested. The graphs depict the time evolution of the ΔF/F after the last spike of each train. *P < 0.05, **P < 0.01, ***P < 0.001. Error bars represent the SEM.

Neuronal firing activates voltage-dependent Ca^2+^ channels. We demonstrated in the previous part of this study that potassium component prevails in the MC AHP and this component is predominantly Ca^2+^-dependent (Figs.4, 3C). Moreover, the Ca^2+^ influx is also responsible for recurrent synaptic transmission in MCs (Isaacson and Strowbridge 1998). Therefore, we wanted to test whether the frequency- and age-dependent modifications of the AHP area could be produce by changes in the Ca^2+^ signal. For this aim, time-lapse imaging was performed on MCs loaded with two different Ca^2+^ probes, the high Ca^2+^ affinity Fluo-4 indicator (P15 *n* = 8; P38 *n* = 6) or the low Ca^2+^ Fluo-4FF indicator (P15 *n* = 6; P38 *n* = 8). As the results obtained with the two probes were not qualitatively different (see Fig. S1) they were grouped together. As exemplified by the neuron shown in Figure6A, MC firing produced a significant increase in the somatic ΔF/F. The quantitative evaluation of the somatic ΔF/F during the few seconds that followed the last spike of each train, that is, during the AHP, showed no difference in the Ca^2+^ response with varying MC firing frequencies (Fig.6B). A similar result was observed when the ΔF/F of the different neurons were pooled together in both the P15 and P38 groups (Fig.6C, *P* > 0.05, paired *t*-test). The absence of modified Ca^2+^ signals for the different firing frequencies was not due to the saturation of the Ca^2+^ probe as a consistent further increase in the ΔF/F was produced by a train of 40 pulses at 20 Hz (Fig.6A). Therefore, the frequency-dependent modification of the AHP area does not seem to be due to a modification of the Ca^2+^ influx during the different firing frequencies. In contrast, we found a significant reduction in the average ΔF/F in the P38 group compared with the P13 for all the firing frequencies tested and for one spike (Fig.7, unpaired *t*-test). This result supports the hypothesis that the reduction in the AHP area in the P38 group is, at least partially, due to a reduction in the AP-induced calcium influx.

### Role of the AHP on MC firing

**Figure 8:**
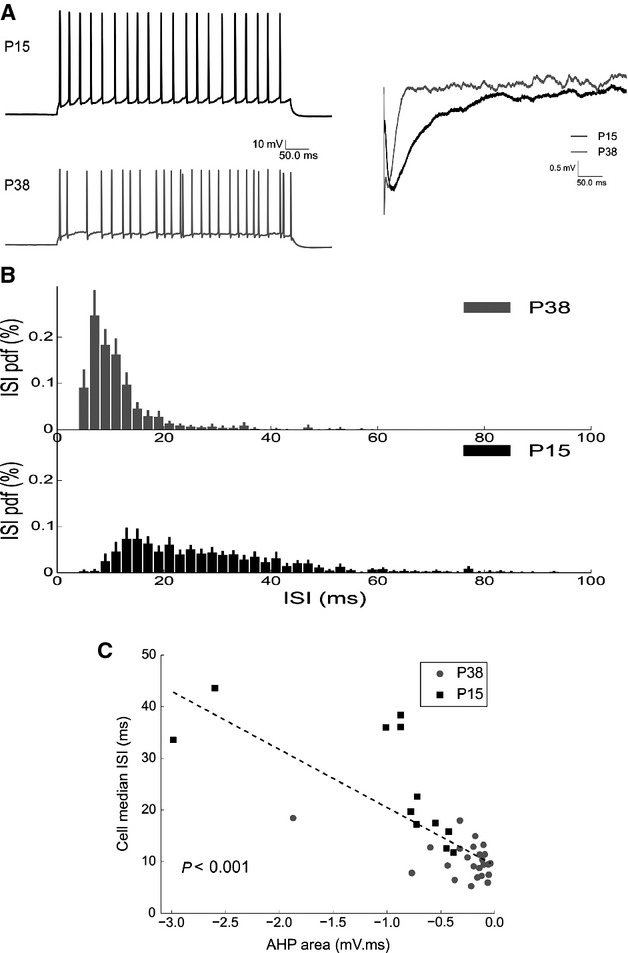
Interspike interval distribution depends on AHP. (A) (left) Representative traces showing the response of MCs from P15 and P38 rats to current steps, (right) average AHP of the same cells following a single spike. (B) Interval interspike distributions computed for each cell and then averaged across cells (error bars are sem, P15: *N* = 16; P38: *N* = 26). Properties of ISI distributions averaged across cells are: for P15 rats, mean: 33 msec, SD: 21 msec; for P38 rats, mean: 12.5 msec, SD: 6.7 msec. Young rats have a slower and more irregular discharge. Pdf = probability density function (C), Median ISI of each cell as a function of cell AHP area (measured after 10 spikes at 20 Hz). Median ISI decreases significantly for larger AHP area (slope: −11.2 mV^−1^, *P* < 0.001, *N* = 37)

**Figure 9:**
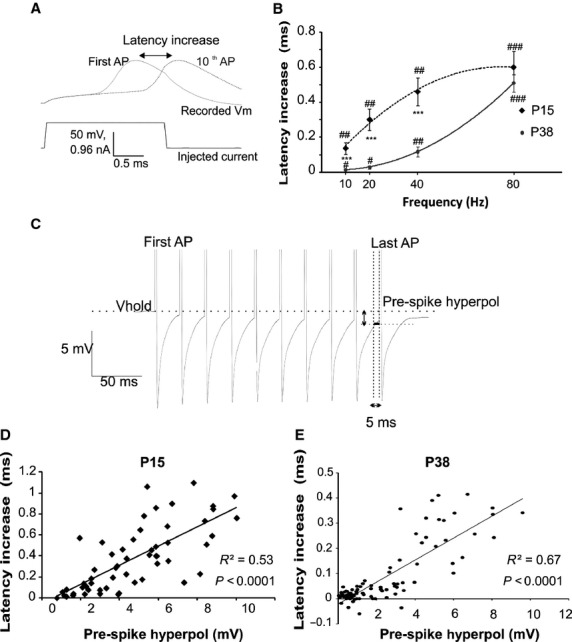
The AHP affects the temporal occurrence of the action potentials during the train. (A) Representative traces showing the latency, from the beginning of the current injection, of the first and the last spike of a train. (B) The effect of the spike train on AP latency increased as a function of the firing frequency in both group of age. Note that latency increase was significantly bigger in the P15 group for all tested MC firing frequencies except 80 Hz. (C) Depict the method used to calculate the prespike hyperpol, that is, the median amplitude of the membrane hyperpolarization (vertical arrow) in the 5 msec preceding the last spike of the train (horizontal arrow). (D and E) The increase in the AP latency correlates with the prespike hyperpol; the data show all prespike hyperpol for all frequencies in P15 D and P38 E groups. *Significantly difference between the two age groups. *P < 0.05; **P < 0.01; ***P < 0.001; Significantly difference between the first and the last spike of each frequency train. ^#^*P* < 0.05; ^##^*P* < 0.01; ^###^*P* < 0.001.

**Figure 10:**
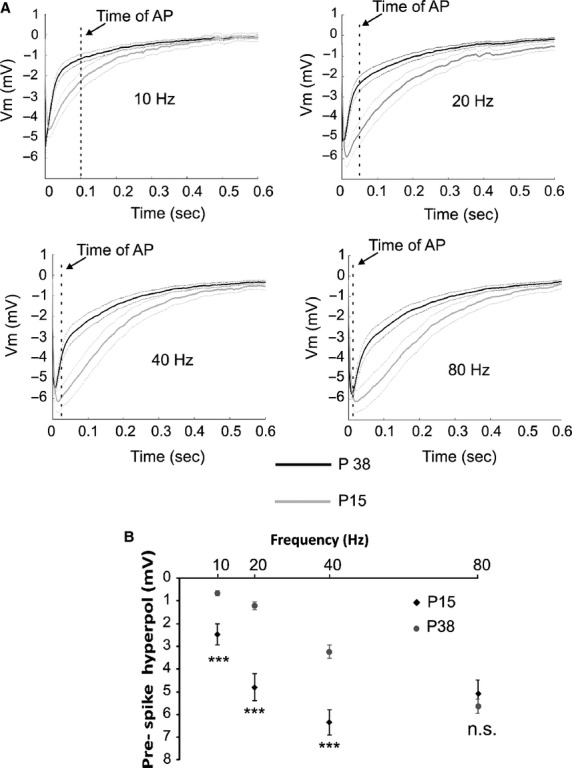
The prespike hyperpol is larger in MC from the P15 groups for all firing frequencies tested except 80 Hz. (A) Comparisons of the average trace of the AHP between the P15 and P38 groups recorded after the last AP of each spike train at different frequencies. Dashed vertical line depicts the occurrence of a putative spike for that specific frequency. Note that at 80 Hz the AHP amplitude at the time of the spike arrival is similar for the two ages. Thin lines represent the SEM. (B) Statistical quantification of the prespike hyperpol for the different frequencies, in the two age group. Significantly difference between the two age groups ***P < 0.001; n.s. not significant.

The membrane hyperpolarization produced by the AHP is proposed to participate in shaping the APs patterning. In order to determine whether and how the AHP impacts upon MC firing we depolarized the neurons with long (500 msec) current injection steps (from rheobase to three times rheobase, Fig.8A). Interspike interval distributions showed a rightward shift in MCs from the P15 group, compared to MCs from P38 animals (Fig.8B), suggesting that larger AHPs are responsible for reduced MC firing frequencies. This was confirmed with a cell-by-cell correlation between MC AHP areas and average firing rates (Fig.8C, Spearman test). During long step depolarization the ISI between the first two APs significantly correlated with the AHP area (*R*^2^ = 0.45, *P* < 0.001, Spearman test; data not shown). Subsequent interspike intervals displayed a spike frequency adaptation which was stronger for higher current steps, and thus for higher firing rates. These observations suggested an impact of the AHP on precise AP timing. To further characterize the mechanisms of AHP action on AP timing spike trains, produced by the protocol using the short (2 msec) current pulses, were analyzed. The spike latency, with respect to the beginning of the depolarizing step, of the first AP of each train with the latency of the last spike of the train, which is potentially affected by the AHP developed during the train (Fig.9A), was compared. For both age groups, we found a significant increase in the latency for the last AP of the train compared with the first for all tested frequencies (Fig.9B, paired *t*-test). This latency difference increases with the MC firing frequency (*P* < 0.001 for both ages; Friedman) and is more important for the P15 group except when MC fires at 80 Hz (groups difference depicted by the asterisks in Fig.9B). The firing-induced modification of AP latency it is unlikely to be produced by a direct effect of the sustained firing on AP generation (for example by inactivation of voltage-dependent Na^+^ channels) because in that case we should have expected a similar spike latency increase in the P15 and P38 groups when stimulated at the same firing frequency. One possibility is that the cause of the observed result is due to the frequency- and age-dependent modification of the MC AHP. However, it is unlikely that the modification of the AP latency is due to the maximal AHP amplitude since, as shown in Figure1E, there are no differences in the maximal AHP amplitude between the two ages despite a difference in spike latency is observed. Moreover, cell-by-cell correlation analysis shows that there is no correlation between the spike latency increase and the maximal AHP amplitude (*R*^2^ = 0.01, data not shown). The determinant parameter could be the amplitude of the AHP at the moment of spike generation. To verify this hypothesis, the level of membrane hyperpolarization relative to the cell Vm and preceding the last spike of the trains (the median Vm during the last 5 msec before the 10th AP), termed prespike hyperpol (Fig.9C) was calculated. As shown in Figure9D and E the prespike hyperpol shows a highly significant correlation with the increase in the spike latency for both the age groups (Pearson test). Interestingly, a much lower correlation index was found when correlating the spike latency increase with the AHP area (*R*^2^ = 0.17, data not shown). The prespike hyperpol hypotheses explain why, at 80 Hz, the increase in spike latency was similar for the P38 and P15 groups. Indeed, as shown in Figure10, at this frequency the prespike hyperpol is similar for the two groups. It should also be noted, from Figure10A, that the spike latency increases cannot be ascribed to the area of the hyperpolarization preceding the last spike. As with increasing frequency the spike comes earlier and earlier in the time course of the preceding AHP, the prespike area is reduced while the spike latency increases. Together these results show that global firing and timing of AP in MC are finely regulated by the AHP, and that the hyperpolarization level reached by the AHP in the few milliseconds preceding an AP influences its timing.

### Olfactory experience affects spike timing by modifying the MC AHP

Neuronal AHP has been shown to be modified following olfactory learning in the piriform cortex (Saar et al. 1998; Saar and Barkai 2009). Moreover, olfactory experience impact MC firing response to odors (Buonviso et al. 1998; Buonviso and Chaput 2000). To determine whether olfactory experience affect MC AHP, the animals (P38 group) were exposed to the odor isoamyl acetate 20 min a day for seven consecutive days. The average V_rest_, AP threshold, membrane resistance, and capacitance were not significantly different for odor-exposed rats compared with the control group (data not shown). In contrast odor exposure produced a reduction in the MC AHP for all the frequencies tested, except 20 Hz, which was limited to the 5–20 msec postspike range (control, *n* = 31; exposed, *n* = 21; unpaired *t*-test; Fig.1). The decrease in the early AHP remained observed in the presence of NBQX APV and gabazine, indicating that this decrease relied on the modification of the potassium component of the AHP (control, *n* = 18; exposed, *n* = 12; *P* < 0.05 unpaired *t*-test; data not shown). This selective AHP reduction was not sufficient to produce significant differences in the AHP amplitude and area in the odor-exposed rats compared with control animals (data not shown). However, the reduction in the early component of the AHP selectively decreases the prespike hyperpolarization only when MCs fire at the higher firing rates, that is, those frequencies for which the AP occurred close or within the first 25 msec of the preceding AHP (40 Hz, 80 Hz, Fig.1B, see also the occurrence of putative AP in Fig.1A). As a consequence, odor exposure impacts spike latency only when MCs fire close to 80 Hz (Fig.1C). Interestingly, during constant current steps injection at different intensities (from 2 times to 4 times rheobase), we observed a tendency of MCs from odor-exposed animals to display shorter ISIs than in control animals (data not shown). Therefore, although the reduction in the early component of the AHP was not dependent on the MC firing frequency, its effect on AP firing was limited to the higher firing frequencies.

**Figure 11:**
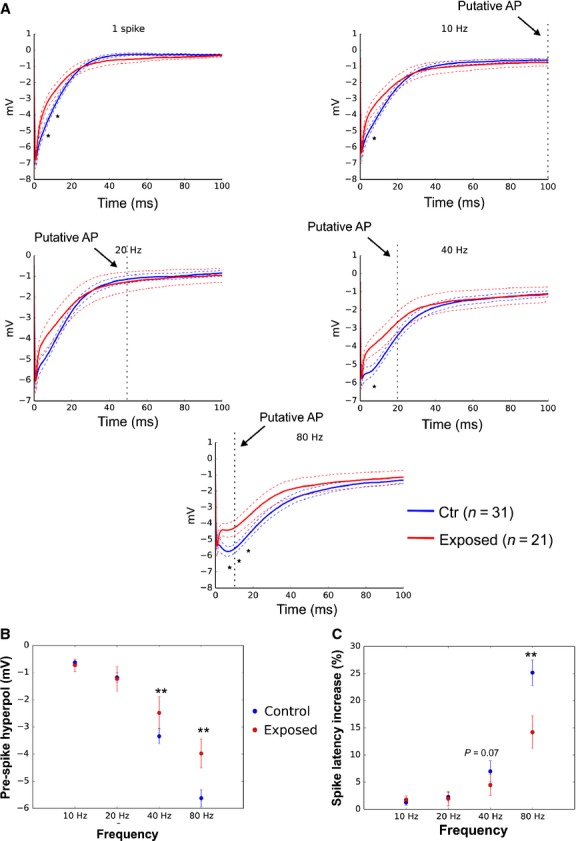
Odor exposure produces a reduction in the MC AHP limited to the range 5–20 msec postspike window. (A) Average traces of the AHP recorded at the end of the spike train in control (blue) and odor-exposed (red) animals. Thin dashed lines represent the SEM. Dashed vertical line depicts the occurrence of a putative spike for that specific frequency. It should be noted that the difference of the AHP between the two group can impact AP only when MC fire at high frequencies (B), The prespike hyperpol is significantly reduced in exposed animals when the MC firing frequencies was higher than 20 Hz. (C) As expected for lower prespike hyperpol the spike latency increase is reduced in exposed animals for frequencies higher than 40 Hz. Significantly difference between the two groups.*P < 0.05; **P < 0.01.

## Discussion

The peculiarity of MC to have a recurrent synaptic transmission has prompted the scientific community to focalize their effort in understanding its cellular mechanisms and the physiological impact exerted by these processes on MC firing. This has led to a large amount of theoretical modeling and speculation on the action of feedback processes on the MC spike coding that, however, is based almost exclusively on the participation of the recurrent DDI and /or the recurrent DDE. In this way, the potential contribution of the co-occurring potassic inhibition has been neglected. Here, we show that the potassium component represents a relevant part of the MC AHP that, at least in juvenile rats, is characterized by a different temporal kinetics than recurrent DDI, suggesting a different impact of the two processes on the control of action potential timing. Such a result should contribute to stimulate a scientific debate on the physiological role of the concomitant feedback processes on MC coding.

We also demonstrate that the contribution of the AHP to the AP latency is neither due the AHP amplitude nor the AHP global area, but to the level of membrane hyperpolarization in the few milliseconds preceding the action potential. This result has important physiological implications showing that the impact of the AHP on action potential generation depends on the combination between the shape of the AHP and the frequency of the inputs generating the AP. In other words, AHP with similar amplitudes or similar area but differing in their decay time will differentially impact AP generation depending on the moment the inputs arrive on the AHP time course. Rapid AHP only affected high-frequency inputs, whereas slow AHP affect a larger range of inputs frequencies. One of the most important consequences is that local modifications of the AHP shape can selectively impact upon spike-inducing inputs that are generated at specific frequencies. Indeed, we showed that olfactory experience, specifically impacts upon high-frequency inputs trough a selective reduction in the early component of the AHP: despite the fact that it does not significantly modify the AHP amplitude or area (Fig.1).

Finally, our results show that MC AHP decreases with the animal age and that such reduction correlates with a reduction in the AP-induced Ca^2+^ signal.”

### Characterization of MC AHP

The mechanisms governing recurrent synaptic transmission have largely been dissected by previous works. Nonetheless, speculations regarding its physiological effect on MC activity are limited by the fact that the different components were studied separately. Moreover, although recurrent synaptic transmission in the MCs is intimately linked to AP generation, the possible contribution of the potassium AHP to the postspike modifications of the membrane potential has generally been neglected. In this study, we demonstrated that the primary postspike feedback component in the MCs is the potassium AHP, which largely determines the inhibition that follows MC firing. This result may appear to contradict a large number of reports showing that recurrent inhibition is primarily or completely blocked by GABA_A_ receptor antagonists. However, the majority of studies regarding recurrent synaptic transmission were performed using a cesium-based solution, thus blocking potassium currents. Indeed, in several current-clamp studies performed with a potassium-based solution, a putative, non-GABAergic hyperpolarizing potassium component of the AHP was presented but not systematically characterized (e.g., see: Friedman and Strowbridge 2000; fig. 6; Salin et al. 2001; figs. 7, 8; Castro et al. 2007, fig. 1; Fukunaga et al. 2012, fig. 4). It has been shown that recurrent DDI can be increased by depolarization-mediated gating of the GC dendritic release of a neurotransmitter (Balu et al. 2007; Arevian et al. 2008; Boyd et al. 2012) possibly through removing a dendritic Mg^2+^ block (Halabisky and Strowbridge 2003; Strowbridge 2009). In our experimental condition, the GC gating effect is most likely underestimated. On the other hand, the effect of the different AHP currents on the MC membrane potential also depends on their driving force. The latter leads us to overestimate the DDI because, in our experimental condition, the V_hold_ was close to the resting membrane potential of the neurons, whereas the imposed reversal potential of the GABA_A_-mediated current (E_Cl_ = −110 mV) was likely more negative than that under physiological conditions (Ben-Ari et al. 2007). Finally, another factor that could affect the quantification of the GABAergic component in the slices could be a partial loss of the MC-GC synapses. Even if they participate to a lesser extent to the global AHP, synaptic components have a significant impact. Interestingly our results indicate that the effect of recurrent DDI in older animals is limited to the early part of the AHP, the first 10–20 msec. As we showed that the impact of the AHP on firing rely on the level of hyperpolarization preceding the AP, this result suggests that, in juvenile but not infant rats, the recurrent DDI would selectively reduce the MC firing for frequency higher than 50 Hz. Such a hypothesis is compatible with a number of reports suggesting that the recurrent DDI participates in gamma oscillatory activity in the OB (Kay et al. 2009). On the other end the reduction in the AHP by the recurrent DDE last several hundred milliseconds, what would impact MC firing for a larger frequency range.

Based on reversal potential and pharmacology our results show that, in the presence of synaptic transmission antagonists, the residual AHP is mainly mediated by potassium currents. However, the contribution of other currents, such as Ih, cannot be excluded. Indeed in the interneurons of the stratum oriens of the hippocampus (Maccaferri and McBain 1996) as well as in the stellate cells of the entorhinal cortex (Nolan et al. 2007), the hyperpolarization produced by the AHP is sufficient to activate the Ih current that, in turn, shapes the AHP by reducing its duration. Despite MCs do express Ih current the V ½ of current activation is around −88 mV (Angelo and Margrie 2011), well more negative than the MCs resting potential (~−62 mV). Therefore, it is likely that the contribution of Ih to MC's AHP is minor.

### Frequency-dependent modification of the MC AHP

We demonstrated that the global MC AHP increases with an increase in the firing rate. In particular, larger AHP areas were produced by increasing the AHP duration. One possibility is that this increase is due to Ca^2+^ accumulation during repetitive firing that could boost both the opening of Ca^2+^-dependent K^+^ channels and recurrent synaptic transmission. However, in this study, we failed to detect any difference in the somatic Ca^2+^ signals produced by the different firing frequencies. This result does not exclude the possibility that a frequency-dependent increase in the intracellular Ca^2+^ concentration could occur on subcellular microdomains during repetitive firing (Berridge 2006), possibly at the level of synaptic and/or K^+^ channels. Alternatively, the frequency-dependent increase in the AHP could rely on the overlap between the inactivation time of the currents participating in the AHP and the MC interspike intervals (Wang et al. 1996).

### Developmental modification of the MC AHP

The results presented in this study demonstrate that the global MC AHP decreases with increasing animal age between P15 and P38. A previous report showed that recurrent DDI decreases with the rat age (Dietz et al. 2011). Here, we extended this finding showing that such a reduction is observed for the three components of the AHP, potassium, recurrent DDI, and recurrent DDE, suggesting that this reduction is due to a common mechanism. We found an increase in membrane resistance that paralleled a decrease in membrane capacitance in the P38 group, which is possibly due to a reduction in MC dendritic arborization during ontogenesis (Malun and Brunjes 1996). Such modifications cannot account for the reduction in the AHP, suggesting that this reduction is likely due to a concomitant reduction in the potassium, GABAergic, and glutamatergic currents linked to AP generation. Our results suggest that one of the mechanisms responsible for the age-dependent decrease in the AHP components is the age-dependent reduction in AP-induced cytosolic Ca^2+^ increase. This reduction could be due to an age-dependent increase in intracellular Ca^2+^ buffering or an increase in intracellular resting Ca^2+^ levels (Neher and Augustine 1992). Alternatively, this reduction could be due to a reduction in voltage-dependent Ca^2+^ currents with increasing age (Schmid and Guenther 1996). In P15 animals, the three components of the AHP similarly increase with the MC firing frequency (Fig.4, Table5), whereas in the P38 group, only the potassium and glutamatergic components do so (Fig.4). At least the following two possible mechanisms could be responsible for the absence of frequency-dependent increases in DDI in P38 animals under our experimental condition: (1) a rapid postsynaptic saturation of GABAergic receptors on the MCs and (2) a rapid ceiling effects of single MC firing to induce GABA release from GC spines. The first hypothesis is quite improbable because this hypothesis would assume that both GC recurrent and lateral inhibition would already be saturated. In contrast, the limitation of GABA release on slices could be enhanced by other mechanisms, such as GC gating during physiological activity. We showed that the decrease in the AHP area and duration in MC from P38, compared with P15, rats produces à shift of the ISI distribution toward lower ISI and reduces the distribution variability (Fig.8). Interestingly such result was forecast by a computational model on the effects of recurrent inhibition on MC ISI distribution (David et al. 2008).

### Experience-dependent modification of the MC AHP

We demonstrated an intrinsic plasticity of the MC AHP produced by odor exposure. The reduction in the AHP was selective of the 5–20 msec postspike period. Such local modification of the AHP clearly affects spike timing selectively for MC firing frequency above 40 Hz (Fig.10), and we were able to put in evidence a tendency for exposed animals to have more ISI below 20 msec compared to control animals. Such slight effect can be due to the small modification of the AHP produced by odor exposure that is not sufficient to reveal substantial modification of the population ISI distribution. Our results show that the MC AHP can be the target of plasticity and open the possibility that the modifications of OB firing activity produced by more consistent olfactory learning paradigms (Ravel et al. 2003) could be, at least in part, mediated by the MC AHP modification. We demonstrate that odor exposure selectively modifies the potassium component of the MC AHP. The characterization of the potassium AHP, primarily in hippocampal neurons, shows the presence of five underling currents (I_M_, I_c_, I_AHP,_ and _s_I_AHP_) that have different temporal and pharmacological profiles (Sah 1996; Gasparini and DiFrancesco 1999; Stocker et al. 1999). A selective odor-induced modification of one or more of these currents is possibly the cause of a selective reduction in the AHP in the 20 msec postspike range observed in this study.

### Functional consequences

Odor information is encoded by spiking activity correlation within specific MC subpopulations (Laurent 2002). In particular, MC synchronization appears to be required to convert the global and noisy odor-evoked firing activity of the MC population in the contrasted and sparse responses observed in the piriform cortex (Laurent 2002; Apicella et al. 2010; Zhan and Luo 2010). Although part of MC synchronization can be imposed by the respiratory rhythm (Margrie and Schaefer 2003; Courtiol et al. 2011; Shusterman et al. 2011; Gerkin et al. 2013), intrinsic properties of MC also appear to regulate the action potential timing within a respiratory cycle (Briffaud et al. 2012). Spike hyper-synchronicity (within <250 *μ*s) between MC pairs was observed in behaving rats performing an olfactory task, and the degree of such synchronization was affected by olfactory learning (Doucette et al. 2011). Due to its capacity to regulate the AP latency within the range of a few hundred *μ*s, the AHP could be one of the mechanisms that, within extrinsically imposed population rhythmicity, would participate in the fine modulation of spike pattering between MCs. The heterogeneity of MC biophysical properties has been shown to affect the degree of correlation between MC population firing and odor coding (Padmanabhan and Urban 2010; Gerkin et al. 2013). We demonstrated that the MC population presents an important heterogeneity of AHP expression and that MC firing frequency correlate with the AHP (Fig.8C, x axe). Therefore, similarities/dissimilarities of the AHP within the MC population could favor the formation of functional neuronal assembles based on correlated firing activity. Moreover, the participation of recurrent synaptic transmission to the MC AHP would potentially cause the AHP, and its effect on MC firing, to be under the influence of neuromodulators or pheromones (Jahr and Nicoll 1982; Otsuka et al. 2001; Ghatpande et al. 2006). Finally, we demonstrated that the MC AHP can be the target of odor-induced plasticity. Therefore, due to its participation in AP timing, the AHP could be involved in the learning-induced modification of MC synchronization observed in this structure.

## Supporting Information

**Figure S1.** Similar profile of AP-induced Ca^2+^ signal obtained using the Ca^2+^ probes Fluo4 and Fluo4-FF.
