# Supplementary figures and images for: Afterhyperpolarization (AHP) regulates the frequency and timing of action potentials in the mitral cells of the olfactory bulb: role of olfactory experience
Source: Physiol Rep. 2015 May 27;3(5):e12344. doi: 10.14814/phy2.12344 (PMC4463813; doi:10.14814/phy2.12344)

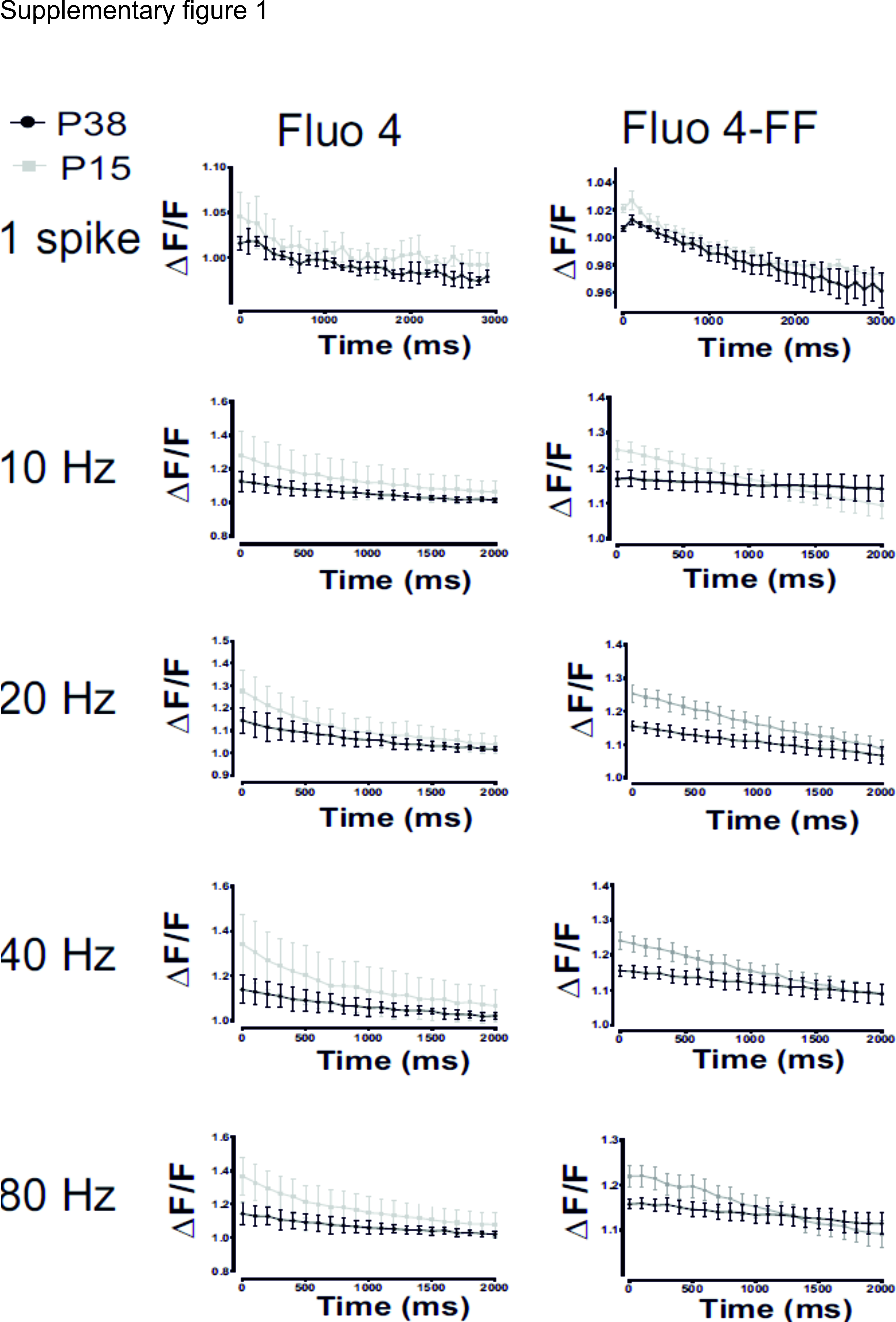

Supplement: Supplementary file 1 [file phy20003-e12344-sd1.tif]
